# Trochlear cartilage status at second‐look arthroscopy predicts 2‐year outcomes after around‐knee osteotomy, especially in older patients

**DOI:** 10.1002/jeo2.70550

**Published:** 2025-11-14

**Authors:** Yuki Kato, Ken Ichikawa, Hiroki Matsui, Shuzo Takazawa, Shin Yamada, Takuya Okada, Akira Ryu, Hiroshi Ohuchi

**Affiliations:** ^1^ Joint Preservation and Reconstruction Center, Kameda Medical Center Kamogawa Chiba Japan; ^2^ Department of Sports Medicine Kameda Medical Center Kamogawa Chiba Japan; ^3^ Clinical Research Support Office, Kameda Medical Center Kamogawa Chiba Japan; ^4^ Department of Clinical Epidemiology and Health Economics, School of Public Health the University of Tokyo Tokyo Japan

**Keywords:** around‐knee osteotomy, knee osteoarthritis, patellofemoral cartilage degeneration, patient‐reported outcomes, second‐look arthroscopy

## Abstract

**Purpose:**

To investigate whether trochlear surface cartilage degeneration of the patellofemoral joint, assessed by second‐look arthroscopy after around‐knee osteotomy (AKO), affects postoperative patient‐reported outcomes and to examine the influence of patient age on this relationship.

**Methods:**

This retrospective cohort study included 331 patients who underwent AKO for varus knee deformity between November 2016 and June 2022. Second‐look arthroscopy was performed approximately 1 year postoperatively, and patients were classified according to femoral trochlear cartilage status at second‐look arthroscopy. International Cartilage Repair Society (ICRS) grades 0–2 were defined as nearly normal and ICRS Grades 3–4 as abnormal for the purpose of this study. The primary outcomes were the Knee Injury and Osteoarthritis Outcome Score (KOOS) and Lysholm score at 2 years. Overlap weighting based on propensity score was used to adjust for confounders.

**Results:**

Of 331 patients, 229 were classified as nearly normal and 102 as abnormal. The abnormal group was older (67.15 ± 8.27 vs. 64.63 ± 9.74 years; *p* < 0.05) and showed persistently lower KOOS Symptoms at 12 and 24 months. In patients ≥65 years, adjusted 24‑month differences favoured the nearly normal group for KOOS Symptoms (−6.83; 95% confidence interval [CI]−12.48 to −1.19). In younger patients, early KOOS symptom deficits resolved by 24 months.

**Conclusions:**

Trochlear cartilage degeneration observed at second‐look arthroscopy after AKO is associated with poorer 2‐year outcomes, particularly in older patients. These findings suggest that a favourable trochlear cartilage status at second‐look is a meaningful prognostic indicator. Monitoring postoperative cartilage status—especially improvement or stability—may aid in identifying patients likely to achieve better recovery and inform age‐specific treatment strategies.

**Level of Evidence:**

Level III.

## Graphical abstract

Trochlear cartilage condition after osteotomy predicts 2‐year outcomes, especially in older patients.

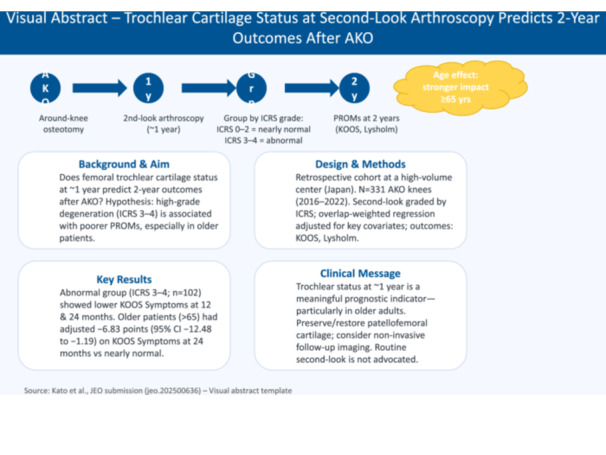

**Abbreviations**
ACI: autologous chondrocyte implantation
ADL: activities of daily living
AKO: around‐the‐knee osteotomy
BMS: bone marrow stimulation
CRA: cartilage repair assessment
DLO: double‐level osteotomy
HKAA: hip–knee–ankle angle
ICRS: International Cartilage Repair Society
JLCA: joint line convergence angle
KL: Kellgren–Lawrence
KOOS: Knee Injury and Osteoarthritis Outcome Score
LCWDFO: lateral closed‐wedge distal femoral osteotomy
mLDFA: mechanical lateral distal femoral angle
mMPTA: mechanical medial proximal tibial angle
MOWHTO: medial open‐wedge high‐tibial osteotomy
OA: osteoarthritis
OAT: osteochondral autograft transfer
OCA: osteochondral allograft transplantation
PROMs: patient‐reported outcome measures
QOL: quality of life
REDCap: Research Electronic Data Capture
%MA: percentage of mechanical axis

## INTRODUCTION

Knee osteoarthritis (OA) is a common and disabling musculoskeletal disorder, especially in individuals over 40 years old [14]. Around‐knee osteotomy (AKO), particularly medial open‐wedge high‐tibial osteotomy (MOWHTO), is an established joint‐preserving surgery for younger or active patients with varus knee OA [9].

Although AKO realigns the tibiofemoral joint, it may increase patellofemoral joint stress, especially in the presence of cartilage damage [1, 6]. Traditionally, severe patellofemoral degeneration has been considered a contraindication for AKO [15]; however, favourable outcomes have been reported even in such cases [2, 10, 11, 12, 20, 22, 32]. Despite this, concerns remain that the surgery itself may accelerate patellofemoral cartilage degeneration postoperatively [3, 17, 20, 27].

Notably, Japan's unique healthcare system and cultural preferences result in routine implant removal approximately 1 year after AKO, regardless of symptoms. Second‐look arthroscopy is commonly performed during the same anaesthesia session, allowing unbiased postoperative evaluation of cartilage status. Supported by universal insurance and practiced widely across institutions, this system reduces selection bias and enhances the generalizability of second‐look findings in this study.

Meanwhile, recent advances in cartilage restoration techniques—such as autologous chondrocyte implantation (ACI), osteochondral autograft transfer (OAT) and osteochondral allograft transplantation (OCA)—have expanded treatment possibilities, even for patellofemoral lesions.

However, prior studies have assessed cartilage status only at the time of AKO, without follow‐up evaluation, leading to uncertainty about its prognostic value [3, 17, 20]. Moreover, it remains unclear how age and activity level influence the relationship between patellofemoral cartilage health and postoperative outcomes, particularly since older adults typically experience lower joint loads during daily activities.

The primary aim of this study was to test this hypothesis by assessing trochlear cartilage status using second‐look arthroscopy approximately 1 year postoperatively and evaluating its relationship with patient‐reported outcomes (PROMs) at 2 years. Because some patients underwent concomitant cartilage repair at the time of AKO, second‐look arthroscopy was also used to evaluate postoperative changes in cartilage condition—including spontaneous deterioration or improvement due to surgery or cartilage repair procedures. A secondary objective was to characterize postoperative changes in cartilage condition in the setting of concomitant cartilage repair at the index surgery. It was hypothesized that trochlear cartilage degeneration observed at second‐look arthroscopy would be associated with poorer PROMs 2 years after AKO, particularly in older patients.

## METHODS

### Study design

This retrospective cohort study used second‐look arthroscopy performed 1 year after AKO as the index date. Data were collected and managed using Research Electronic Data Capture (REDCap), a secure, web‐based software platform designed to support data capture for research studies [13]. An in‐house case registry was constructed in REDCap, which encompassed all patients who underwent surgery at the institution. For cases treated prior to REDCap implementation, relevant data from electronic medical records were retrospectively entered into the registry. Trained medical personnel reviewed the data and manually transferred the necessary information. Only data from the REDCap‐based registry were used in the present study.

### Setting

This study was conducted at a high‐volume general hospital in Japan that serves as a regional centre for orthopaedic surgery, including trauma and sports medicine.

AKO for varus knee deformity is usually reserved for relatively young, active patients with medial knee OA or subchondral insufficiency fracture of the knee (SIFK). At the institution, older adult patients who remain active and do not have severe comorbidities were also considered candidates.

At the time of the initial surgery, which involved AKO, diagnostic arthroscopy (first‐look arthroscopy) was routinely performed to evaluate the intra‐articular environment and determine the appropriate surgical strategy, including cartilage repair procedures, when necessary. A standardized follow‐up protocol was implemented after AKO. Patients were seen in the outpatient setting every 3 months during the first postoperative year, every 6 months during the second year, and annually thereafter. Regardless of the occurrence of adverse events, metal implant removal and second‐look arthroscopy are routinely performed after confirming osteotomy site healing, typically at least 1 year postsurgery.

### Surgical technique and rehabilitation

Knee osteotomies were performed for varus knee deformity associated with OA, SIFK or traumatic cartilage injuries. The procedures included MOWHTO, medial open‐wedge distal tuberosity osteotomy (MOWDTO), and double‐level osteotomy (DLO), defined as a combination of lateral closed‐wedge distal femoral osteotomy (LCWDFO) with either MOWHTO or MOWDTO. Lateral closed‐wedge high‐tibial osteotomy (LCWHTO) was not performed at the institution during the study period and was not included.

The index procedure was selected primarily according to the planned correction angle (MOWHTO < 8°, MOWDTO 8 – <14°, DLO ≥ 14°), while the centre of rotation of the angulation and radiographic parameters (e.g., mLDFA and mMPTA) were also considered. In selected patients with severe varus deformity, less invasive procedures (MOWHTO or MOWDTO alone) were chosen when advanced age, comorbidities or limited tolerance to highly invasive surgery were present.

Concomitant cartilage procedures were performed as indicated: autologous chondrocyte implantation (ACI) using JACC® (autologous cultured cartilage; Japan Tissue Engineering Co., Ltd.) [21] for large defects (≥ 4 cm²), osteochondral autograft transplantation (OAT) for localized lesions, and bone marrow stimulation (BMS) when other options were not feasible. For intra‐articular procedures, including cartilage repair, a vertical skin incision was made using either a midvastus or subvastus approach. The patella was laterally retracted to expose the knee joint. Patellar tracking was assessed intraoperatively and lateral retinacular release was performed when necessary.

Postoperative rehabilitation followed an institutional protocol. The weight‐bearing protocol was advanced from toe‐touch after drain removal, to 1/3 partial weight‐bearing on postoperative Day 15, and full weight‐bearing was targeted from the fourth week. For patients who underwent concomitant cartilage repair of the femoral trochlea, an extension‐locked brace was worn for three months to ensure weight‐bearing in full extension.

### Study population

Consecutive knees (*n* = 427) undergoing AKO for varus knee OA at the institution between November 2016 and June 2022 were screened for eligibility according to predefined criteria (see Eligibility criteria).

### Eligibility criteria (inclusion and exclusion)

Inclusion: Consecutive knees undergoing AKO for varus knee OA at the institution between November 2016 and June 2022 were eligible if a second‑look arthroscopy at implant removal was performed at approximately 1 year postoperatively and before the 2‑year PROMs assessment to preserve temporal sequence.

**Figure 1:**
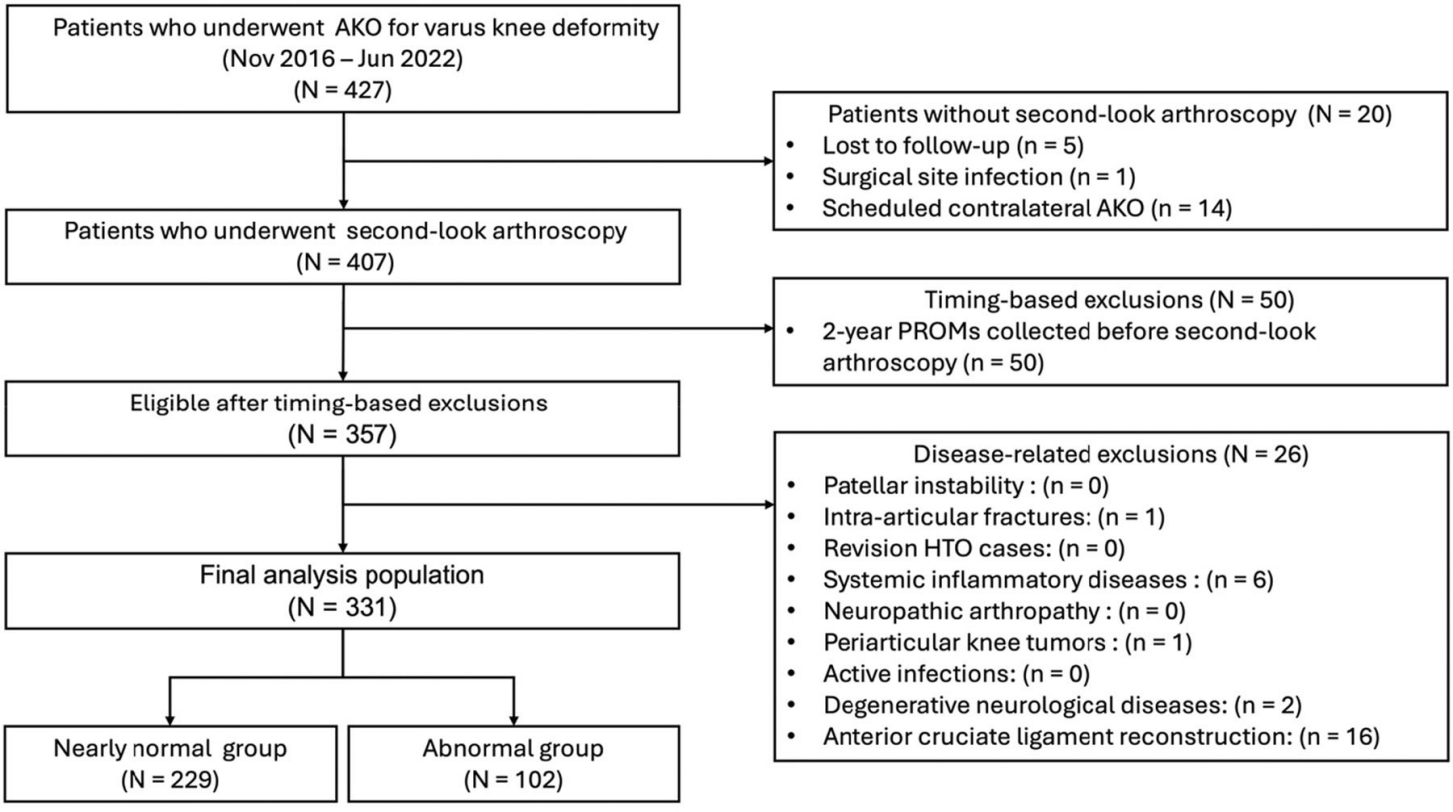
Flowchart of patient selection. AKO, around‐knee osteotomy; HTO, high‐tibial osteotomy; PROMs, patient‐reported outcomes.

Exclusion: Knees were excluded if the second‑look was not performed within the study schedule (e.g., loss to follow‑up, postoperative infection precluding implant removal or staged contralateral AKO delaying the second‑look), if 2‑year PROMs had been obtained before the second‑look, or if predefined disease‑related conditions were present (intra‑articular fracture, systemic inflammatory disease, periarticular knee tumour, degenerative neurological disease or prior anterior cruciate ligament reconstruction). Patient flow is summarized in Figure 1.

### Study size

The sample size was determined based on the total number of eligible patients at the institution during the study period. As this was an observational study, all patients who met the inclusion criteria during the study period were included.

### Exposure

At the second‑look arthroscopy approximately 1 year postoperatively, the femoral trochlea of the knee joint was evaluated using the International Cartilage Repair Society (ICRS) classification system [4]. In this study, the nearly normal group was defined as knees with femoral trochlear cartilage ICRS grades 0–2 (normal–mild degeneration) at second‑look arthroscopy, whereas the abnormal group was defined as ICRS grades 3–4 (severe degeneration).

### Outcomes and study variables

The primary outcome measures were PROMs, specifically the Knee Injury and Osteoarthritis Outcome Score (KOOS) and the Lysholm score, assessed 2 years postoperatively. PROMs were assessed using the Japanese‑validated KOOS and the Lysholm Knee Scoring Scale. The KOOS Japanese version has undergone formal cross‑cultural adaptation and psychometric validation [23]. The Lysholm scale, originally developed for knee ligament injuries, has well‑established psychometric properties; a Japanese‑language validation study has not been identified to our knowledge, so Lysholm was used as a secondary/supportive measure [26].

Patient demographic data, including age (dichotomized at 65 years: ≤65 and >65 years), sex, height, weight, body mass index and Charlson Comorbidity Index, were extracted from electronic medical records. In addition, surgical details were collected encompassing AKO types, namely MOWHTO, MOWDTO, DLO and LCWDFO, and information on concomitant procedures and cartilage repair procedures and period from initial surgery to second‐look arthroscopy. The cartilage damage status was evaluated based on arthroscopic findings at the time of osteotomy. To evaluate typical cartilage degeneration, the ICRS classification (Grades 0–4) was used, whereas areas treated with cartilage repair procedures were assessed using the ICRS cartilage repair assessment (ICRS CRA grade, Grades 1–4).

The radiographic measurements were performed by an independent examiner who was not involved in the surgical procedure. Preoperative and 12‐month postoperative full‐length anteroposterior radiographs of the lower extremity were used to obtain the following parameters: Kellgren–Lawrence (KL) classification, hip–knee–ankle angle (HKAA), percentage of mechanical axis (%MA), joint line convergence angle (JLCA), mechanical medial proximal tibial angle (mMPTA), mechanical lateral distal femoral angle (mLDFA), Insall–Salvati ratio, patellar tilt angle and sulcus angle. Among the various radiographic parameters, the HKAA, %MA, JLCA, mMPTA and mLDFA were measured using mediCAD Classic (Toyo Technica), a digital orthopaedic planning software package. The remaining parameters were evaluated using SYNAPSE (Fujifilm), a filmless picture archiving and communication system. These long‐leg alignment measurements—including those performed with mediCAD—have shown good‐to‐excellent inter‐ and intra‐observer reliability in prior studies [18].

### Statistical analysis

Descriptive statistics were computed for patient characteristics in both groups. Univariate analyses were subsequently performed to compare group characteristics. Continuous variables were analysed using *t*‐tests, and categorical variables were analysed using *χ* ^2^ tests. All statistical analyses were performed using R (version 4.3.3, R Foundation for Statistical Computing). PROMs were not available in a small proportion of cases: 1.2% preoperatively, 0.6% at 12 months and 12.1% at 24 months postoperatively. These missing data were not imputed; instead, available‐case analysis was applied. Patients with missing PROMs were not lost to follow‐up overall, as clinical care continued beyond the missing time points.

Supporting Information S1: Figure 1 displays the directed acyclic graph hypothesized in this study to represent causal relationships among the variables. In this investigation, exposure was defined as the status of patellofemoral cartilage degeneration observed during second‐look arthroscopy performed at least 1 year postoperatively, while the outcome was the treatment result measured using clinical scores. We hypothesized that trochlear repair and lateral release could be confounding factors. Additionally, the achievement of postoperative valgus alignment, as determined by the HKAA, was considered an important confounder. The correction angle may also introduce bias owing to its effects on patellar height and postoperative physical activity. Age was also identified as a potential confounding variable. Accordingly, the following variables were incorporated as independent factors in the propensity score model: presence of trochlear repair, lateral release, postoperative knee alignment (categorized into four groups based on the %MA: <50%, 50%–57%, 57%–67% and ≥67%), postoperative mechanical medial proximal tibial angle (categorized into three groups: <90°, 90°–95° and ≥95°), and age, categorized into three groups for adjustment only (<60, 60–70 and ≥70 years) based on the directed acyclic graph‑informed propensity‑score model.

Overlap weighting was applied using the calculated propensity scores to adjust for imbalances in confounding factors between the groups. After weighting, a linear regression model was used to estimate the differences in outcomes between the groups, with the exposure variable as the independent variable. The model included age (categorized), body mass index (BMI), type of osteotomy, lateral release, postoperative alignment (%MA and mMPTA) and the presence of trochlear cartilage repair. Baseline trochlear status based on intraoperative ICRS grading was also incorporated. Thus, the overlap‐weighted regression analysis allowed us to estimate the independent association between trochlear degeneration and PROMs. Robust sandwich variance estimators were employed to calculate the variance in the estimates, as overlap weighting can alter the error structure. This method provides valid standard errors even when conventional variance estimation may be biased due to heteroskedasticity or weighting‐induced misspecification.

### Stratified analysis

To examine the potential differences in the relationship between patellofemoral cartilage degeneration and PROMs by age, a stratified analysis was conducted. The patients were divided into two groups: those aged 65 years or younger and those older than 65 years; 65 years was selected as the stratification threshold as it is commonly used in orthopaedic research to distinguish between younger and older adults and reflects the physiological and functional differences associated with aging. A recent systematic review reported that 65 years of age was the most frequently used cut‐off for age‐based stratification in orthopaedic studies, being used in 27.2% of the included articles [12]. These groups are referred to throughout the manuscript as the younger group (≤65 years) and the older group (>65 years). The same statistical models and variables used in the primary analysis were also used in the stratified analysis.

## RESULTS

Participants and baseline characteristics. During the study period, 427 AKO knees were screened. Of these, 407 (95.3%) underwent second‑look arthroscopy at implant removal, performed at a mean of 437.7 (Standard Deviation: 73.7) days postoperatively. Twenty knees without second‑look arthroscopy were excluded (loss to follow‑up, *n* = 5; postoperative infection precluding implant removal, *n* = 1; planned staged contralateral AKO, *n* = 14). An additional 50 knees were excluded because 2‑year PROMs were obtained before the second‑look, and 26 were excluded due to predefined disease‑related criteria. The final analysis included 331 knees, classified as nearly normal (ICRS: 0–2; *n* = 229) or abnormal (ICRS: 3–4; *n* = 102) based on femoral trochlear status at the second‑look. Figure 1 shows the patient‑selection flow, and Table 1 summarizes baseline characteristics.

**Table 1:** Patient demographics, surgical procedures and preoperative PROMs in the nearly normal and abnormal groups.

	Nearly normal group (*N* = 229)	Abnormal group (*N* = 102)	*p* value	SMD
Age (years, mean (SD))	64.63 (9.74)	67.15 (8.27)	0.024	0.278
Age ≤ 65 (*n* (%))	105 (45.9)	43 (42.2)	0.614	0.074
Sex (male) (*n* (%))	82 (35.8)	24 (23.5)	0.037	0.271
Body mass index (mean (SD))	25.20 (2.87)	25.77 (3.43)	0.118	0.18
Height (mean (SD))	1.60 (0.09)	1.58 (0.09)	0.206	0.152
Weight (mean (SD))	64.51 (10.63)	64.75 (10.69)	0.854	0.022
CCI (mean (SD))	0.21 (0.52)	0.24 (0.45)	0.721	0.044
AKO type			0.403	0.209
MOWHTO (*n* (%))	157 (68.6)	76 (74.5)		
MOWDTO (*n* (%))	35 (15.3)	9 (8.8)		
DLO (LCWDFO‐MOWHTO) (*n* (%))	22 (9.6)	9 (8.8)		
DLO (LCWDFO‐MOWDTO) (*n* (%))	15 (6.6)	8 (7.8)		
Concomitant surgical procedures				
Lateral retinacular release (*n* (%))	161 (70.3)	60 (58.8)	0.055	0.242
Cruciate ligament reconstruction (*n* (%))	0 (0)	0 (0)	N/A	0
Meniscectomy (*n* (%))	132 (57.6)	64 (62.7)	0.453	0.104
Meniscal repair (*n* (%))	2 (0.9)	1 (1.0)	1	0.011
Cartilage repair methods				
Bone marrow stimulation (*n* (%))	167 (72.9)	84 (82.4)	0.087	0.228
Osteochondral autograft transfer (*n* (%))	211 (92.1)	96 (94.1)	0.681	0.078
Autologous chondrocyte implantation (*n* (%))	21 (9.2)	2 (2.0)	0.032	0.318
Period from Initial surgery to second‐look arthroscopy (days) (mean (SD))	437.86 (73.73)	438.05 (73.80)	0.982	0.003
Chondral defect location at first‐look arthroscopy (ICRS Grade ≥ 3)				
MFC (*n* (%))	216 (94.3)	100 (98.0)	0.224	0.195
MTP (*n* (%))	174 (76.0)	92 (90.2)	0.004	0.386
LFC (*n* (%))	16 (7.0)	23 (22.5)	<0.001	0.45
LTP (*n* (%))	2 (0.9)	9 (8.8)	0.001	0.377
Patella (*n* (%))	53 (23.1)	48 (47.1)	<0.001	0.518
Trochlea (*n* (%))	137 (59.8)	79 (77.5)	0.003	0.387
Chondral defect location at second‐look arthroscopy (ICRS Grade ≥ 3)				
MFC (*n* (%))	81 (35.4)	56 (54.9)	0.001	0.4
MTP (*n* (%))	121 (52.8)	70 (68.6)	0.01	0.328
LFC (*n* (%))	5 (2.2)	6 (5.9)	0.161	0.189
LTP (*n* (%))	4 (1.7)	9 (8.8)	0.006	0.32
Patella (*n* (%))	32 (14.0)	50 (49.0)	<0.001	0.815
Trochlea (*n* (%))	0 (0.0)	102 (100.0)	<0.001	NaN
Preoperative PROMs				
KOOS subscales				
Pain (mean (SD))	61.47 (18.11)	57.59 (17.98)	0.074	0.215
Symptoms (mean (SD))	65.39 (18.90)	59.26 (18.55)	0.007	0.327
ADL (mean (SD))	76.24 (15.43)	73.68 (14.56)	0.158	0.171
Sports (mean (SD))	38.03 (25.36)	34.45 (25.97)	0.244	0.14
QOL (mean (SD))	38.91 (21.88)	38.06 (18.99)	0.737	0.041
Lysholm Score (mean (SD))	63.76 (19.66)	59.33 (17.99)	0.059	0.235

Abbreviations: ADL, activities of daily living; AKO, around‐knee osteotomy; CCI, Charlson's Comorbidity Index; DLO, double‐level osteotomy; ICRS, International Cartilage Repair Society; KOOS, Knee Injury and Osteoarthritis Outcome Score; LFC, lateral femoral condyle; LCWDFO, lateral closing wedge distal femoral osteotomy; LTP, lateral tibial plateau; MOWHTO, medial opening wedge high‐tibial osteotomy; MOWDTO, medial opening wedge distal tuberosity osteotomy; MFC, medial femoral condyle; MTP, medial tibial plateau; NaN, Not a Number; PROMs, patient‐reported outcome measures; QOL, quality of life; SD, standard deviation; SMD, standardized mean difference.

Compared with the nearly normal group, the abnormal group was older on average (67.15 vs. 64.63 years) and showed a higher frequency of preoperative high‑grade cartilage lesions across compartments; the abnormal group also had a lower proportion of males and fewer MOWDTO cases, while other baseline differences were modest (Table 1). Covariate balance was improved after overlap weighting (Supporting Information S1: Table 1).

Table 2 summarizes radiographic parameters at both preoperative and 12‐month postoperative time points. KL Grade 4 was more common in the abnormal group (43.1% vs. 23.1%, *p* = 0.001). The sulcus angle was slightly smaller in the abnormal group (139.32° vs. 137.68 °, *p* = 0.016). At 12 months, both groups showed valgus correction, with significant changes in HKAA, %MA and JLCA. However, no significant differences were observed in patellar tilt angle or Insall–Salvati ratio, indicating limited change in patellofemoral alignment.

**Table 2:** Radiographic parameters before and 12 months after surgery in the nearly normal and abnormal groups.

	Nearly normal group (*N* = 229)	Abnormal group (*N* = 102)	*p* value	SMD
Preoperative Radiographic Parameters				
KL classification			<0.001	0.511
0	0 (0.0)	0 (0.0)		
1	9 (3.9)	3 (2.9)		
2	61 (26.6)	15 (14.7)		
3	111 (48.5)	40 (39.2)		
4	48 (21.0)	44 (43.1)		
HKAA (degree, mean (SD))	−7.24 (4.01)	−8.25 (4.11)	0.036	0.249
%MA (%, mean (SD))	17.34 (15.87)	13.68 (15.69)	0.053	0.232
JLCA (degree, mean (SD))	3.23 (2.15)	3.88 (2.39)	0.014	0.287
mMPTA (degree, mean (SD))	83.99 (2.94)	83.69 (2.73)	0.38	0.106
mLDFA (degree, mean (SD))	87.92 (2.09)	88.22 (2.37)	0.241	0.136
Insall‐Salvati ratio (mean (SD))	0.99 (0.15)	1.01 (0.16)	0.381	0.105
Patellar Tilt Angle (degree, mean (SD))	7.55 (3.75)	6.98 (3.60)	0.201	0.155
Sulcus Angle (degree, mean (SD))	139.32 (4.60)	137.68 (7.43)	0.016	0.266
12‐month postoperative radiographic parameters				
HKAA (degree, mean (SD))	2.82 (2.69)	2.12 (2.78)	0.033	0.254
%MA (%, mean (SD))	60.41 (10.54)	57.58 (11.10)	0.027	0.262
JLCA (degree, mean (SD))	2.63 (1.98)	3.14 (2.24)	0.036	0.244
mMPTA (degree, mean (SD))	92.73 (2.72)	93.13 (2.79)	0.217	0.147
mLDFA (degree, mean (SD))	87.33 (2.43)	87.83 (2.85)	0.103	0.189
Insall‐Salvati ratio (mean (SD))	0.99 (0.15)	1.01 (0.16)	0.381	0.105
Patellar Tilt Angle (degree, mean (SD))	5.42 (3.40)	5.45 (4.14)	0.949	0.008

Abbreviations: %MA, percentage of mechanical axis; HKAA, hip–knee–ankle angle; JLCA, joint line convergence angle; KL, Kellgren and Lawrence classification; mMPTA, mechanical medial proximal tibial angle; mLDFA, mechanical lateral distal femoral angle; SMD, standardized mean difference; SD, standard deviation.

Table 3 presents the clinical scores at 12 and 24 months after surgery. At 12 months postoperatively, the abnormal group showed lower scores, with KOOS Symptoms and sports being significantly lower than those of the nearly normal group. At 24 months postoperatively, the abnormal group continued to show significantly lower KOOS Symptom scores than did the nearly normal group.

**Table 3:** KOOS and Lysholm scores at 12 and 24 months postoperatively in the nearly normal and abnormal groups.

	Nearly normal group (*N* = 229)	Abnormal group (*N *= 102)	*p* value
12‐month postoperative PROMs			
KOOS Subscales			
Pain (mean (SD))	83.03 (12.67)	80.26 (15.18)	0.086
Symptoms (mean (SD))	81.12 (12.88)	75.25 (17.68)	0.001
ADL (mean (SD))	88.53 (10.11)	87.00 (11.17)	0.218
Sports (mean (SD))	57.29 (24.27)	49.66 (28.37)	0.013
QOL (mean (SD))	65.97 (20.13)	65.07 (21.62)	0.716
Lysholm score (mean (SD))	85.09 (12.19)	80.40 (17.72)	0.006
24‐month postoperative PROMs			
KOOS Subscales			
Pain (mean (SD))	85.43 (13.19)	82.77 (13.14)	0.115
Symptoms (mean (SD))	82.78 (12.72)	78.73 (15.23)	0.02
ADL (mean (SD))	90.73 (9.57)	88.69 (10.16)	0.101
Sports (mean (SD))	63.03 (26.94)	59.83 (27.03)	0.353
QOL (mean (SD))	71.64 (20.22)	71.38 (19.78)	0.917
Lysholm Score (mean (SD))	86.33 (11.76)	84.51 (14.86)	0.267

Abbreviations: ADL, activities of daily living; KOOS, Knee Injury and Osteoarthritis Outcome Score; PROMs, patient‐reported outcome measures, QOL, quality of life; SD, standard deviation.

Table 4 presents the differences in outcomes between the groups, along with their confidence intervals, both before and after adjustment. In both unadjusted and adjusted analyses, the point estimates for all outcomes were lower in the abnormal group than in the nearly normal group. For the KOOS Symptoms, the confidence intervals did not cross zero in either the crude or adjusted analyses. Table 5 summarizes PROMs stratified by age (>65 vs. ≤65 years) and the presence or absence of trochlear cartilage degeneration. In the older group, no baseline differences were observed. However, at 12 months, patients with cartilage degeneration showed significantly lower Lysholm (84.07 vs. 78.51, *p* = 0.022) and KOOS Symptoms (79.46 vs. 74.27, *p* = 0.023) scores. These differences persisted at 24 months for KOOS Pain (85.19 vs. 80.17, *p* = 0.025) and KOOS Symptoms (82.81 vs. 76.75, *p* = 0.009). In the younger group, the abnormal group had significantly lower KOOS Symptoms (83.11 vs. 76.58, *p* = 0.015) at 12 months. By 24 months, these differences were no longer significant. Overall, older patients with cartilage degeneration exhibited persistent deficits in pain and symptoms, while younger patients recovered by 2 years.

**Table 4:** Unadjusted and adjusted differences in KOOS and Lysholm Scores between the nearly normal and abnormal groups.

	Unadjusted score difference (95% CI)	Adjusted score difference (95% CI)
KOOS subscales		
Pain	−2.66 (−5.97, 0.64)	−2.61 (−6.30, 1.08)
Symptoms	−4.04 (−7.44, −0.64)	−4.44 (−8.49, −0.39)
ADL	−2.04 (−4.50, 0.40)	−2.00 (−4.90, 0.90)
Sports	−3.20 (−9.97, 3.57)	−4.02 (−11.39, 3.35)
QOL	−0.27 (−5.31, 4.78)	−1.23 (−6.84, 4.39)
Lysholm Score	−1.81 (−5.02, 1.40)	−2.34 (−6.33, 1.65)

Abbreviations: ADL, activities of daily living; CI, confidence interval; KOOS, Knee Injury and Osteoarthritis Outcome Score; QOL, quality of life.

**Table 5:** Age‐stratified comparison of KOOS and Lysholm Scores between the nearly normal and abnormal groups.

	Older group (age > 65)			Younger group (age ≤ 65)		
	Nearly normal group (*N* = 117)	Abnormal group (*N *= 59)	*p* value	Nearly normal group (*N* = 102)	Abnormal group (*N* = 50)	*p* value
Preoperative PROMs						
KOOS subscales						
Pain (mean (SD))	60.89 (17.72)	58.52 (18.51)	0.411	62.36 (18.60)	56.33 (17.37)	0.071
Symptoms (mean (SD))	66.32 (19.35)	60.34 (17.97)	0.049	64.50 (18.35)	57.81 (19.43)	0.051
ADL (mean (SD))	74.98 (15.37)	73.15 (14.04)	0.444	77.75 (15.52)	74.38 (15.36)	0.233
Sports (mean (SD))	35.04 (22.96)	35.09 (25.78)	0.99	41.67 (27.78)	33.57 (26.51)	0.11
QOL (mean (SD))	39.74 (22.47)	40.95 (19.06)	0.723	37.99 (21.32)	34.08 (18.38)	0.3
Lysholm Score (mean (SD))	62.54 (20.43)	59.71 (17.74)	0.375	65.42 (18.71)	58.80 (18.53)	0.06
12‐Month Postoperative PROMs						
KOOS subscales						
Pain (mean (SD))	81.97 (13.42)	78.58 (15.26)	0.129	84.30 (11.62)	82.56 (14.95)	0.45
Symptoms (mean (SD))	79.46 (13.22)	74.27 (16.50)	0.023	83.11 (12.22)	76.58 (19.32)	0.015
ADL (mean (SD))	86.80 (10.75)	84.27 (11.47)	0.147	90.62 (8.90)	90.73 (9.68)	0.946
Sports (mean (SD))	52.74 (24.95)	46.27 (28.99)	0.122	62.77 (22.34)	54.30 (27.14)	0.052
QOL (mean (SD))	65.57 (19.50)	64.51 (21.35)	0.739	66.44 (20.95)	65.84 (22.22)	0.877
Lysholm Score (mean (SD))	84.07 (13.38)	78.51 (18.44)	0.022	86.33 (10.48)	83.00 (16.53)	0.147
24‐Month Postoperative PROMs						
KOOS subscales						
Pain (mean (SD))	85.19 (12.75)	80.17 (13.99)	0.025	85.71 (13.79)	86.34 (11.08)	0.808
Symptoms (mean (SD))	82.81 (12.55)	76.75 (15.49)	0.009	82.73 (13.00)	81.47 (14.62)	0.631
ADL (mean (SD))	89.61 (9.91)	86.56 (10.40)	0.075	92.11 (9.00)	91.61 (9.18)	0.777
Sports (mean (SD))	59.60 (26.99)	55.00 (28.04)	0.321	67.25 (26.41)	66.49 (24.41)	0.879
QOL (mean (SD))	72.10 (18.74)	68.75 (20.77)	0.308	71.09 (22.00)	75.00 (17.98)	0.339
Lysholm Score (mean (SD))	85.70 (12.67)	82.12 (16.32)	0.13	87.10 (10.57)	87.81 (12.03)	0.741

Abbreviations: ADL, activities of daily living; KOOS, Knee Injury and Osteoarthritis Outcome Score; QOL, quality of life.

Table 6 summarizes the impact of femoral trochlear cartilage degeneration (ICRS: Grade 3–4) on 2‐year KOOS subscales and Lysholm scores, stratified by age. In the younger cohort (≤65 years), the unadjusted model showed significantly lower KOOS Symptoms in the abnormal group, but this difference was not significant after adjustment. In contrast, the adjusted model in the older cohort (>65 years) revealed significantly lower Symptoms (−6.83) in the abnormal group. Although differences in Pain, ADL, Sports, QOL and Lysholm were not statistically significant, all estimates favoured the nearly normal group. Adjusted baseline characteristics after overlap weighting are shown in Supporting Information S1: Tables 2 and 3.

**Table 6:** Unadjusted and adjusted differences in 2‐Year PROMs stratified by age group ( ≤ 65 vs. >65 Years).

	Older group (age > 65)		Younger group (age ≤ 65)	
	Unadjusted Score Difference (95% CI)	Adjusted Score Difference (95% CI)	Unadjusted Score Difference (95% CI)	Adjusted Score Difference (95% CI)
KOOS subscales				
Pain	−2.66 (−5.97, 0.64)	−4.67 (−9.87, 0.53)	−2.66 (−5.97, 0.65)	−0.89 (−5.43, 3.66)
Symptoms	−4.40 (−7.44, −0.64)	−6.83 (−12.48, −1.19)	−4.40 (−7.44, −0.64)	−1.90 (−7.70, 3.92)
ADL	−2.05 (−4.50, 0.40)	−3.15 (−6.99, 0.68)	−2.05 (−4.99, 0.40)	−1.08 (−4.93, 2.76)
Sports	−3.20 (−9.97, 3.57)	−8.22 (−17.99, 1.55)	−3.20 (−9.97, 3.57)	−1.69 (−12.22, 8.84)
QOL	−0.27 (−5.31, 4.78)	−3.63 (−11.38, 4.11)	−0.27 (−5.31, 4.78)	0.32 (−7.65, 8.28)
Lysholm score	−1.81 (−5.02, 1.40)	−3.67 (−9.50, 2.16)	−1.81 (−5.02, 1.40)	−1.25 (−6.61, 4.10)

Abbreviations: ADL, activities of daily living; CI, confidence interval; KOOS, Knee Injury and Osteoarthritis Outcome Score; QOL, quality of life.

## DISCUSSION

The main finding of this study is that femoral trochlear cartilage degeneration, as observed during second‐look arthroscopy 1 year after AKO, was significantly associated with worse PROMs at 2 years postoperatively, particularly in the KOOS Symptoms subscale; knees without trochlear degeneration had more favourable prognoses. The association was stronger among older patients, suggesting that patellofemoral, and particularly trochlear, degeneration contributes substantially to postoperative functional decline after AKO, especially in elderly individuals.

Reports on patellofemoral degeneration after AKO are conflicting: several studies describe progression [27, 28, 31], whereas others suggest little change or stability [11, 20, 32]. To our knowledge, no prior work has linked postoperative cartilage status after AKO to later PROMs or shown a greater impact in older adults. By assessing cartilage status at 1 year and relating it to 2‑year PROMs, the present study clarifies this relationship and provides evidence useful for intermediate evaluation and the development of therapeutic targets.

In the present study, the effect of joint degeneration on postoperative PROMs was more pronounced in older patients. Age modified the association between degeneration and outcomes. A plausible explanation is diminished neuromuscular reserve with aging [5] and greater susceptibility to activity‑related pain and progressive OA changes [16, 24]. Younger individuals may compensate for joint imbalance, whereas this capacity is reduced in older adults. Although the younger cohort showed no 2‑year deficit attributable to trochlear degeneration, delayed effects may emerge as physiological reserves decline; longitudinal follow‑up is warranted.

In routine AKO management, patellofemoral cartilage is often deemphasized because several studies reported minimal influence on postoperative outcomes [2, 10, 11, 20, 22, 32]. The present results indicate that patellofemoral cartilage status at approximately 1 year may affect subsequent PROMs in older patients, supporting postoperative monitoring of patellofemoral degeneration. Routine second‐look arthroscopy is not advocated; where intermediate assessment is needed, noninvasive methods such as quantitative MRI may be preferable, although further validation is required [25].

Age‐related physiology should inform surgical indications. In older adults with advanced patellofemoral degeneration—where cartilage restoration is technically challenging or unlikely to succeed—osteotomy may warrant reconsideration in favour of total knee arthroplasty. Although adjunctive cartilage‐restoration procedures during AKO have been reviewed [19], most evidence centres on BMS and rarely addresses patellofemoral lesions; effects on PROMs remain uncertain. In this cohort, a favourable cartilage repair appearance at 1 year coincided with better outcomes; however, causality cannot be inferred and overstatement should be avoided. Priority should be given to identifying patients most likely to benefit from cartilage repair in the AKO setting; achieving high success—even in older patients—will require meticulous technique and further research, including for extensive, nonfocal lesions. Preoperative rehabilitation aimed at soft‑tissue function may help limit postoperative degeneration. Although ΔICRS values were not analysed formally, descriptive data (Table 1) suggest improvement from Grade ≥3 to ≤2 in some knees within the nearly normal group, with minimal change in the abnormal group, consistent with persistent/progressive damage. Biomechanical literature underscores the importance of trochlear integrity for patellofemoral mechanics; dysplasia alters tracking, increases contact pressures and reduces stability [8, 30], supporting the present focus on the trochlear.

This study has several strengths. Second‐look arthroscopy approximately 1 year, provided direct, objective assessment of postoperative cartilage status; the sample size was relatively large (*n* = 331) with comprehensive radiographic, arthroscopic and clinical data; and overlap‑weighted analyses reduced confounding. The 2‐year follow‐up and age‐stratified analyses highlighted a greater impact of cartilage degeneration in older patients. Although arthroscopic ICRS grading system is subjective, it is widely used; dichotomization into Grades 0–2 versus 3–4 reflects clinically meaningful thresholds and may enhance reproducibility in the trochlea, with acceptable reliability reported previously [7, 29]. These features support the validity and practicality of the present stratification.

Limitations should be acknowledged. Postoperative lower limb muscle strength was not assessed, despite its relevance to recovery in older patients [16]. The single‐centre observational design limits generalizability and warrants multicenter [2, 20]. Inclusion of multiple AKO techniques introduced heterogeneity; correction angles can influence patellofemoral joint cartilage degeneration [27], and some knees did not achieve optimal alignment, although models adjusted for tibial correction angle, HKAA and Insall–Salvati ratio. KOOS at 1 year was not included as a covariate, assuming it does not affect cartilage status at that time, which is an inherent limitation of observational designs. Longitudinal change in trochlear status (ΔICRS) and MRI‑based baseline staging were unavailable; baseline arthroscopic status was incorporated, but residual confounding from subclinical degeneration or postoperative progression cannot be excluded. Finally, sample size‐limited mediation analyses to separate direct from indirect effects. Larger multicenter studies with serial MRI and formal mediation are needed.

## CONCLUSION

Trochlear cartilage condition approximately 1 year after AKO was associated with PROMs at 2 years. Poor cartilage status at this stage, especially involving the femoral trochlea, was linked to worse outcomes in older patients. While intermediate imaging or arthroscopy is not routinely required, aiming for a favourable patellofemoral cartilage condition within the first postoperative year may support better functional recovery. In selected patients, cartilage repair performed during AKO could contribute to improved prognosis, but careful case‐by‐case decision‐making remains crucial.

## AUTHOR CONTRIBUTIONS

The corresponding author conceived and designed the study, performed all surgical procedures, acquired and managed the data, drafted the manuscript, and approved the final version. He takes full responsibility for the integrity and accuracy of the data and manuscript. Another author contributed to data collection and management and assisted with patient support and data entry. Statistical analyses were conducted by a third author, who also guided data interpretation and critically reviewed the manuscript. The remaining coauthors assisted with data acquisition and surgical procedures and contributed to manuscript revision. All authors reviewed and approved the final manuscript.

## CONFLICT OF INTEREST STATEMENT

The authors declare no other conflicts of interest.

## ETHICS STATEMENT

This study was approved by the Institutional Review Board of our institution (approval number: 24‐074, Date: September 27th, 2024). Informed consent was obtained through an opt‐out process by providing an opt‐out form on the website; patients who refused to participate were excluded from the study. Informed consent was obtained through an opt‐out process by providing an opt‐out form on the website; patients who refused to participate were excluded from the study.

## Supporting information

Supporting information.

## Data Availability

All data used in this study are stored securely within the authors’ institution and are not publicly available due to data protection policies. However, all coauthors had full access to the dataset.

## References

[1] Astur DC , Arliani GG , Binz M , Astur N , Kaleka CC , Amaro JT , et al. Autologous osteochondral transplantation for treating patellar chondral injuries: evaluation, treatment, and outcomes of a two‐year follow‐up study. J Bone Joint Surg Am. 2014;96(10):816–823. 24875022 10.2106/JBJS.M.00312

[2] Berend KR , Lombardi, Jr. AV , Morris MJ , Hurst JM , Kavolus JJ . Does preoperative patellofemoral joint state affect medial unicompartmental arthroplasty survival? Orthopedics. 2011;34(9):e494–e496. 21902142 10.3928/01477447-20110714-39

[3] Bin SI , Kim HJ , Ahn HS , Rim DS , Lee DH . Changes in patellar height after opening wedge and closing wedge high tibial osteotomy: a meta‐analysis. Arthroscopy. 2016;32(11):2393–2400. 27570171 10.1016/j.arthro.2016.06.012

[4] Brittberg M , Winalski CS . Evaluation of cartilage injuries and repair. J Bone Joint Surg Am. 2003;85–A(Suppl 2):58–69. 10.2106/00004623-200300002-00008 12721346

[5] Chen L , Zhou H , Gong Y , Tang Y , Su H , Jin Z , et al. How do muscle function and quality affect the progression of KOA? A narrative review. Orthop Surg. 2024;16(4):802–810. 38438160 10.1111/os.14022 PMC10984828

[6] Duncan RC , Hay EM , Saklatvala J , Croft PR . Prevalence of radiographic osteoarthritis‐‐it all depends on your point of view. Rheumatology. 2006;45(6):757–760. 16418199 10.1093/rheumatology/kei270

[7] Dwyer T , Martin CR , Kendra R , Sermer C , Chahal J , Ogilvie‐Harris D , et al. Reliability and validity of the arthroscopic international cartilage repair society classification system: correlation with histological assessment of depth. Arthroscopy. 2017;33(6):1219–1224. 28162918 10.1016/j.arthro.2016.12.012

[8] Feller JA , Amis AA , Andrish JT , Arendt EA , Erasmus PJ , Powers CM . Surgical biomechanics of the patellofemoral joint. Arthroscopy. 2007;23(5):542–553. 17478287 10.1016/j.arthro.2007.03.006

[9] Floerkemeier S , Staubli AE , Schroeter S , Goldhahn S , Lobenhoffer P . Outcome after high tibial open‐wedge osteotomy: a retrospective evaluation of 533 patients. Knee Surg Sports Traumatol Arthrosc. 2013;21(1):170–180. 22744433 10.1007/s00167-012-2087-2

[10] Goshima K , Sawaguchi T , Horii T , Shigemoto K , Iwai S , Hatsuchi Y . Patellofemoral osteoarthritis progression after open‐wedge high tibial osteotomy does not affect the clinical outcomes or survivorship at minimum 7 years’ follow‐up. Arthroscopy. 2024;40(1):93–102. 37209776 10.1016/j.arthro.2023.05.007

[11] Goshima K , Sawaguchi T , Shigemoto K , Iwai S , Nakanishi A , Ueoka K . Patellofemoral Osteoarthritis progression and alignment changes after open‐wedge high tibial osteotomy do not affect clinical outcomes at mid‐term follow‐up. Arthroscopy. 2017;33(10):1832–1839. 28633973 10.1016/j.arthro.2017.04.007

[12] Gupta NK , Dunivin F , Chmait HR , Smitterberg C , Buttar A , Fazal‐Ur‐Rehman M , et al. Orthopedic frailty risk stratification (OFRS): a systematic review of the frailty indices predicting adverse outcomes in orthopedics. J Orthop Surg. 2025;20(1):247. 10.1186/s13018-025-05609-2 PMC11887260 40051013

[13] Harris PA , Taylor R , Thielke R , Payne J , Gonzalez N , Conde JG . Research electronic data capture (REDCap)‐‐a metadata‐driven methodology and workflow process for providing translational research informatics support. J Biomed Inf. 2009;42(2):377–381. 10.1016/j.jbi.2008.08.010 PMC2700030 18929686

[14] Hatzikotoulas K , Southam L , Stefansdottir L , Boer CG , McDonald ML , Pett JP , et al. Translational genomics of osteoarthritis in 1,962,069 individuals. Nature. 2025;641(8065):1217–1224. 40205036 10.1038/s41586-025-08771-z PMC12119359

[15] Insall J , Shoji H , Mayer V . High tibial osteotomy. A five‐year evaluation. J Bone Jt Surg. 1974;56(7):1397–1405. 4433363

[16] Javadian Y , Adabi M , Heidari B , Babaei M , Firouzjahi A , Ghahhari BY , et al. Quadriceps muscle strength correlates with serum vitamin d and knee pain in knee osteoarthritis. Clin J Pain. 2017;33(1):67–70. 26889621 10.1097/AJP.0000000000000358

[17] Javidan P , Adamson GJ , Miller JR , Durand Jr. P , Dawson PA , Pink MM , et al. The effect of medial opening wedge proximal tibial osteotomy on patellofemoral contact. Am J Sports Med. 2013;41(1):80–86. 23108639 10.1177/0363546512462810

[18] Jud L , Roth T , Fürnstahl P , Vlachopoulos L , Sutter R , Fucentese SF . The impact of limb loading and the measurement modality (2D versus 3D) on the measurement of the limb loading dependent lower extremity parameters. BMC Musculoskelet Disord. 2020;21(1):418. 32605616 10.1186/s12891-020-03449-1 PMC7329436

[19] Kahlenberg CA , Nwachukwu BU , Hamid KS , Steinhaus ME , Williams 3rd RJ . Analysis of outcomes for high tibial osteotomies performed with cartilage restoration techniques. Arthroscopy. 2017;33(2):486–492. 27773639 10.1016/j.arthro.2016.08.010

[20] Kataoka K , Watanabe S , Nagai K , Kay J , Matsushita T , Kuroda R , et al. Patellofemoral osteoarthritis progresses after medial open‐wedge high tibial osteotomy: a systematic review. Arthroscopy. 2021;37(10):3177–3186. 33895305 10.1016/j.arthro.2021.04.015

[21] Kato Y , Yamada S , Takazawa S , Hattori S , Okada T , Ohuchi H . Comparative study on clinical outcomes in autologous chondrocyte implantation using three‐dimensional cultured JACC® with collagen versus periosteum coverings. Sci Rep. 2024;14(1):9834. 38684723 10.1038/s41598-024-59798-7 PMC11058265

[22] Lee YS , Lee SB , Oh WS , Kwon YE , Lee BK . Changes in patellofemoral alignment do not cause clinical impact after open‐wedge high tibial osteotomy. Knee Surg Sports Traumatol Arthrosc. 2016;24(1):129–133. 25288336 10.1007/s00167-014-3349-y

[23] Nakamura N , Takeuchi R , Ishikawa H , Saito T , Sawaguchi T , Goldhahn S . Cross‐cultural adaptation and validation of the Japanese Knee Injury and Osteoarthritis Outcome Score (KOOS). J Orthop Sci. 2011;16(5):516–523. 21766211 10.1007/s00776-011-0112-9

[24] Patterson BE , Girdwood MA , West TJ , Bruder AM , Øiestad BE , Juhl C , et al. Muscle strength and osteoarthritis of the knee: a systematic review and meta‐analysis of longitudinal studies. Skeletal Radiol. 2023;52(11):2085–2097. 36562820 10.1007/s00256-022-04266-4

[25] Shinohara M , Akagi R , Watanabe A , Kato Y , Sato Y , Morikawa T , et al. Time‐dependent change in cartilage repair tissue evaluated by magnetic resonance imaging up to 2 years after atelocollagen‐assisted autologous cartilage transplantation: data from the CaTCh study. Cartilage. 2022;13(3):194760352211092. 10.1177/19476035221109227 PMC9277438 35815923

[26] Smith HJ , Richardson JB , Tennant A . Modification and validation of the Lysholm Knee Scale to assess articular cartilage damage. Osteoarthr Cartil. 2009;17(1):53–58. 10.1016/j.joca.2008.05.002 18556222

[27] Song SJ , Yoon KH , Park CH . Patellofemoral cartilage degeneration after closed‐ and open‐wedge high tibial osteotomy with large alignment correction. Am J Sports Med. 2020;48(11):2718–2725. 32762564 10.1177/0363546520943872

[28] Tanaka T , Matsushita T , Miyaji N , Ibaraki K , Nishida K , Oka S , et al. Deterioration of patellofemoral cartilage status after medial open‐wedge high tibial osteotomy. Knee Surg Sports Traumatol Arthrosc. 2019;27(4):1347–1354. 30196435 10.1007/s00167-018-5128-7

[29] van den Borne MPJ , Raijmakers NJH , Vanlauwe J , Victor J , de Jong SN , Bellemans J , et al. International Cartilage Repair Society (ICRS) and Oswestry macroscopic cartilage evaluation scores validated for use in Autologous Chondrocyte Implantation (ACI) and microfracture. Osteoarthr Cartil. 2007;15(12):1397–1402. 10.1016/j.joca.2007.05.005 17604187

[30] Van Haver A , De Roo K , De Beule M , Labey L , De Baets P , Dejour D , et al. The effect of trochlear dysplasia on patellofemoral biomechanics: a cadaveric study with simulated trochlear deformities. Am J Sports Med. 2015;43(6):1354–1361. 25740833 10.1177/0363546515572143

[31] Yoon TH , Choi CH , Kim SJ , Kim SH , Kim NH , Jung M . Effect of medial open‐wedge high tibial osteotomy on the patellofemoral joint according to postoperative realignment. Am J Sports Med. 2019;47(8):1863–1873. 31157981 10.1177/0363546519851096

[32] Yoon WK , Kim KI , Kim JH , Lee SH , Jo MG . Does degeneration of the patellofemoral joint after medial open‐wedge high tibial osteotomy affect clinical outcomes? Am J Sports Med. 2022;50(11):2972–2979. 35914309 10.1177/03635465221113324

