# Supplementary material for: Trochlear cartilage status at second‐look arthroscopy predicts 2‐year outcomes after around‐knee osteotomy, especially in older patients
Source: J Exp Orthop. 2025 Nov 14;12(4):e70550. doi: 10.1002/jeo2.70550 (PMC12616396; doi:10.1002/jeo2.70550)
Supplement: Supplementary file 1 — Supporting information. [file JEO2-12-e70550-s001.docx]

**Online Resource 1**


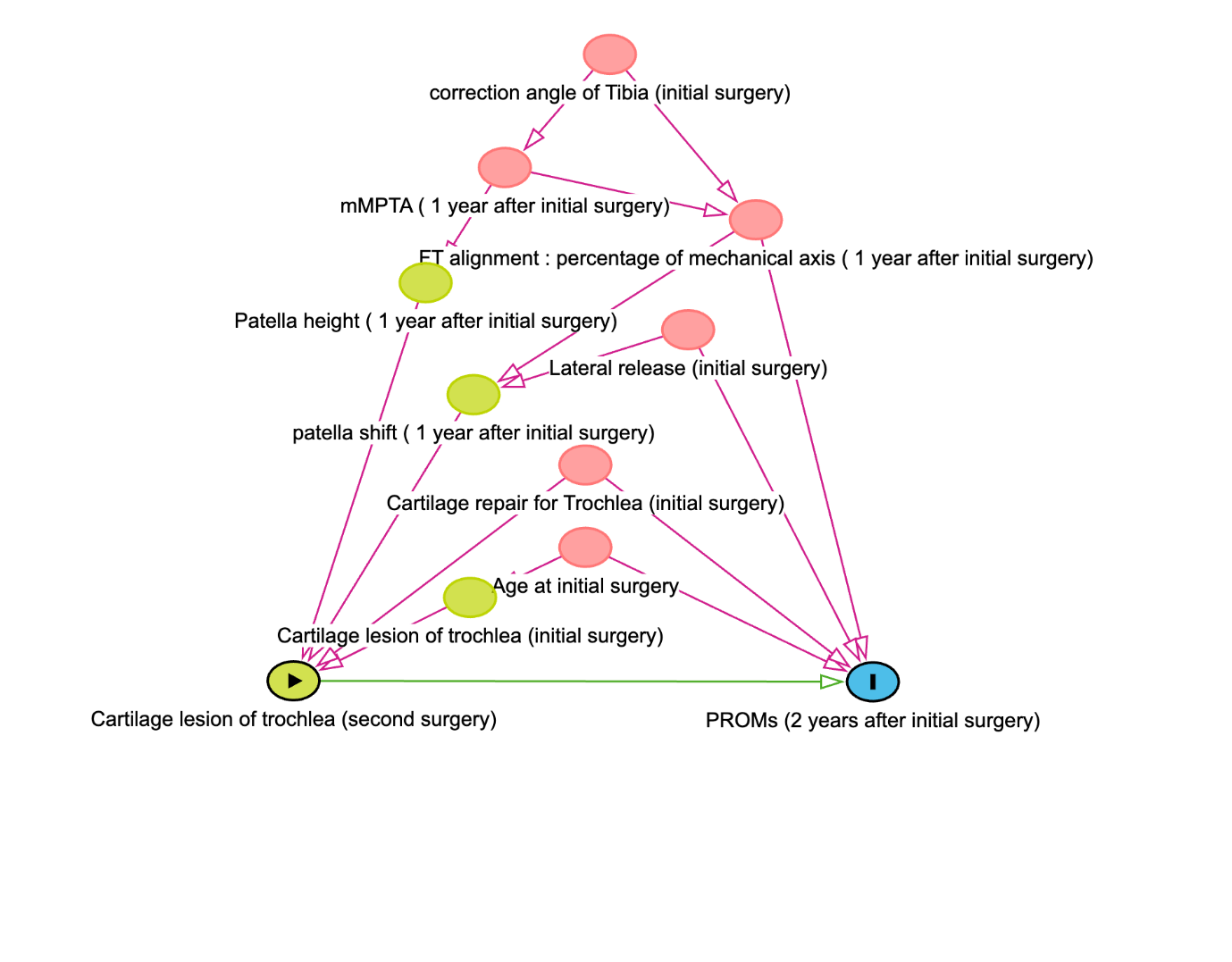


## Supplementary Figure 1. Causal model depicting the relationship between trochlear cartilage degeneration and postoperative outcomes following osteotomy around the knee. Directed acyclic graph (DAG) illustrating the assumed relationships among variables influencing the association between trochlear cartilage degeneration and postoperative patient-reported outcomes (PROMs) following around-knee osteotomy (AKO). “Initial surgery” refers to the index AKO procedure during which first-look arthroscopy is performed. “Second-look arthroscopy” was conducted approximately 12 months postoperatively, coinciding with implant removal. The DAG included potential confounders, such as age, sex, BMI, and baseline PROMs. Overlap weighting using a propensity score was performed to adjust for confounders in the statistical analysis. Patella-related biomechanical parameters, such as the Insall–Salvati ratio (patellar height), patellar tilt angle, and sulcus angle, were not included as direct covariates in the statistical model. However, their biomechanical effects were accounted for indirectly by adjusting for upstream variables—such as correction angle, type of osteotomy, and postoperative alignment (e.g., %MA and mMPTA)—which influence patellofemoral joint loading and mechanics. These upstream determinants were incorporated into the propensity score model to ensure adequate control for their potential confounding influence.

## mMPTA, mechanical Medial Proximal Tibial Angle; FT, Femorotibial Joint; PROMs, Patient-Reported Outcome Measures

## **Online Resource 2**

## Supplementary Table 1. Adjusted baseline characteristics after overlap weighting: early normal vs. abnormal (Full Cohort)

|  | Nearly Normal Group (N = 61.29) | Abnormal Group (N = 61.29) | P-value | SMD |
| --- | --- | --- | --- | --- |
| Age (years, mean (SD)) | 66.65 (8.73) | 66.62 (8.53) | 0.978 | 0.003 |
| Sex (male) (n (%)) | 19.8 (32.3) | 15.3 (25.0) | 0.217 | 0.163 |
| Body Mass Index (mean (SD)) | 25.06 (2.75) | 25.67 (3.41) | 0.131 | 0.195 |
| Height (mean (SD)) | 1.59 (0.08) | 1.59 (0.09) | 0.902 | 0.016 |
| Weight (mean (SD)) | 63.22 (9.89) | 64.76 (10.83) | 0.248 | 0.149 |
| CCI (mean (SD)) | 0.19 (0.49) | 0.21 (0.43) | 0.754 | 0.037 |
| AKO Type |  |  | 0.471 | 0.203 |
| MOWHTO (n (%)) | 44.9 (73.2) | 43.1 (70.3) |  |  |
| MOWDTO (n (%)) | 8.3 (13.5) | 6.8 (11.0) |  |  |
| DLO (LCWDFO-MOWHTO) (n (%)) | 5.0 (8.2) | 6.0 (9.8) |  |  |
| DLO (LCWDFO-MOWDTO) (n (%)) | 3.1 (5.0) | 5.4 (8.8) |  |  |
| Concomitant Surgical Procedures |  |  |  |  |
| Lateral Retinacular Release (n (%)) | 39.0 (63.7) | 39.0 (63.7) | 1 | 0 |
| Cruciate Ligament Reconstruction (n (%)) | 0 (0%) | 0 (0%) | N/A | 0 |
| Meniscectomy (n (%)) | 34.8 (56.8) | 39.1 (63.8) | 0.262 | 0.144 |
| Meniscal Repair (n (%)) | 0.7 (1.1) | 0.5 (0.8) | 0.803 | 0.032 |
| Cartilage Repair Methods |  |  |  |  |
| Bone Marrow Stimulation (n (%)) | 40.6 (66.2) | 50.3 (82.0) | 0.007 | 0.368 |
| Osteochondral Autograft Transfer System (n (%)) | 56.8 (92.7) | 57.8 (94.2) | 0.631 | 0.061 |
| Autologous Chondrocyte Implantation (n (%)) | 2.0 (3.3) | 1.7 (2.8) | 0.828 | 0.03 |
| Period from Initial Surgery to Second-Look Arthroscopy (days) (mean (SD)) | 430.11 (61.91) | 438.58 (74.04) | 0.325 | 0.124 |
| Chondral Defect Location at First-look Arthroscopy (ICRS Grade ≥ 3) |  |  |  |  |
| MFC (n (%)) | 57.9 (94.5) | 60.2 (98.2) | 0.122 | 0.202 |
| MTP (n (%)) | 46.3 (75.6) | 54.6 (89.1) | 0.012 | 0.359 |
| LFC (n (%)) | 2.3 (3.8) | 14.3 (23.4) | <0.001 | 0.595 |
| LTP (n (%)) | 0.2 (0.4) | 6.3 (10.2) | <0.001 | 0.448 |
| Patella (n (%)) | 11.7 (19.1) | 29.5 (48.1) | <0.001 | 0.645 |
| Trochlea (n (%)) | 24.7 (40.2) | 48.9 (79.8) | <0.001 | 0.882 |
| Chondral Defect Location at second-look Arthroscopy (ICRS Grade ≥ 3) |  |  |  |  |
| MFC (n (%)) | 19.7 (32.1) | 31.8 (52.0) | 0.002 | 0.41 |
| MTP (n (%)) | 32.4 (52.8) | 40.2 (65.6) | 0.048 | 0.262 |
| LFC (n (%)) | 0.8 (1.3) | 4.1 (6.7) | 0.008 | 0.282 |
| LTP (n (%)) | 0.7 (1.1) | 6.1 (10.0) | <0.001 | 0.394 |
| Patella (n (%)) | 5.7 (9.3) | 30.5 (49.7) | <0.001 | 0.988 |
| Trochlea (n (%)) | 0.0 (0.0) | 61.3 (100.0) | <0.001 | NaN |
| Pre-Operative PROMs |  |  |  |  |
| KOOS subscales |  |  |  |  |
| Pain (mean (SD)) | 60.58 (17.75) | 57.41 (18.32) | 0.176 | 0.176 |
| Symptoms (mean (SD)) | 65.13 (18.99) | 59.09 (18.94) | 0.014 | 0.319 |
| ADL (mean (SD)) | 74.75 (15.05) | 73.67 (15.07) | 0.582 | 0.072 |
| Sports (mean (SD)) | 37.72 (25.45) | 34.63 (25.90) | 0.351 | 0.121 |
| QOL (mean (SD)) | 39.04 (22.60) | 37.57 (18.98) | 0.576 | 0.07 |
| Lysholm Score (mean (SD)) | 62.23 (20.21) | 59.10 (18.04) | 0.212 | 0.163 |

SMD, Standardized Mean Difference; SD, Standard Deviation; CCI, Charlson’s Comorbidity Index; AKO, Around-Knee Osteotomy; MOWHTO, Medial Opening Wedge High-Tibial Osteotomy; MOWDTO, Medial Opening Wedge Distal Tuberosity Osteotomy; DLO, Double-Level Osteotomy; LCWDFO, Lateral Closing Wedge Distal Femoral Osteotomy; ICRS, International Cartilage Repair Society; MFC, Medial Femoral Condyle; MTP, Medial Tibial Plateau; LFC, Lateral Femoral Condyle; LTP, Lateral Tibial Plateau; PROMs, Patient-Reported Outcome Measures; KOOS, Knee Injury and Osteoarthritis Outcome Score; ADL, Activities of Daily Living; QOL, Quality of Life

## **Online Resource 3**

## Supplementary Table 2. Adjusted baseline characteristics after overlap weighting: younger patients (≤65 Years)

|  | Nearly Normal Group (N = 24.67) | Abnormal Group (N = 24.67) | P-value | SMD |
| --- | --- | --- | --- | --- |
| Age (years, mean (SD)) | 57.97 (6.02) | 58.44 (5.03) | 0.671 | 0.084 |
| Sex (male) (n (%)) | 9.2 (37.4) | 6.6 (27.0) | 0.287 | 0.226 |
| Body Mass Index (mean (SD)) | 25.78 (2.57) | 26.22 (4.23) | 0.543 | 0.126 |
| Height (mean (SD)) | 1.62 (0.09) | 1.61 (0.08) | 0.71 | 0.075 |
| Weight (mean (SD)) | 67.55 (10.15) | 67.85 (11.49) | 0.891 | 0.028 |
| CCI (mean (SD)) | 0.15 (0.42) | 0.16 (0.37) | 0.877 | 0.029 |
| AKO Type |  |  | 0.238 | 0.339 |
| MOWHTO (n (%)) | 19.7 (79.8) | 18.8 (76.4) |  |  |
| MOWDTO (n (%)) | 3.9 (16.0) | 3.1 (12.6) |  |  |
| DLO (LCWDFO-MOWHTO) (n (%)) | 1.0 (4.0) | 2.7 (11.0) |  |  |
| DLO (LCWDFO-MOWDTO) (n (%)) | 0.1 (0.2) | 0.0 (0.0) |  |  |
| Concomitant Surgical Procedures |  |  |  |  |
| Lateral Retinacular Release (n (%)) | 12.2 (49.3) | 12.2 (49.3) | 1 | 0 |
| Cruciate Ligament Reconstruction (n (%)) | 0 (0%) | 0 (0%) | N/A | <0.001 |
| Meniscectomy (n (%)) | 10.6 (42.9) | 14.3 (57.9) | 0.137 | 0.305 |
| Meniscal Repair (n (%)) | 0.1 (0.3) | 0.0 (0.0) | 0.328 | 0.078 |
| Cartilage Repair Methods |  |  |  |  |
| Bone Marrow Stimulation (n (%)) | 14.0 (56.6) | 18.5 (75.1) | 0.062 | 0.398 |
| Osteochondral Autograft Transfer System (n (%)) | 20.7 (83.9) | 23.9 (96.9) | 0.064 | 0.455 |
| Autologous Chondrocyte Implantation (n (%)) | 2.1 (8.6) | 1.5 (5.9) | 0.638 | 0.102 |
| Period from Initial Surgery to Second-Look Arthroscopy (days) (mean (SD)) | 424.59 (59.07) | 423.04 (59.34) | 0.894 | 0.026 |
| Chondral Defect Location at First-look Arthroscopy (ICRS Grade ≥ 3) |  |  |  |  |
| MFC (n (%)) | 22.3 (90.3) | 23.3 (94.6) | 0.441 | 0.165 |
| MTP (n (%)) | 16.5 (66.9) | 21.8 (88.3) | 0.024 | 0.533 |
| LFC (n (%)) | 1.5 (6.2) | 6.1 (24.7) | 0.005 | 0.527 |
| LTP (n (%)) | 0.1 (0.4) | 2.5 (10.1) | <0.001 | 0.45 |
| Patella (n (%)) | 4.0 (16.2) | 10.4 (42.3) | 0.003 | 0.599 |
| Trochlea (n (%)) | 9.8 (39.7) | 20.6 (83.5) | <0.001 | 1.008 |
| Chondral Defect Location at second-look Arthroscopy (ICRS Grade ≥ 3) |  |  |  |  |
| MFC (n (%)) | 5.8 (23.6) | 12.3 (49.7) | 0.006 | 0.562 |
| MTP (n (%)) | 8.9 (36.2) | 16.0 (64.7) | 0.005 | 0.595 |
| LFC (n (%)) | 0.4 (1.8) | 0.6 (2.4) | 0.804 | 0.042 |
| LTP (n (%)) | 0.3 (1.2) | 1.6 (6.5) | 0.045 | 0.281 |
| Patella (n (%)) | 2.6 (10.6) | 9.6 (39.1) | <0.001 | 0.697 |
| Trochlea (n (%)) | 0.0 (0.0) | 24.7 (100.0) | <0.001 | NaN |
| Pre-Operative PROMs |  |  |  |  |
| KOOS subscales |  |  |  |  |
| Pain (mean (SD)) | 61.40 (17.65) | 56.70 (17.93) | 0.194 | 0.264 |
| Symptoms (mean (SD)) | 64.14 (17.20) | 58.03 (19.88) | 0.11 | 0.329 |
| ADL (mean (SD)) | 76.41 (14.78) | 74.33 (16.25) | 0.523 | 0.134 |
| Sports (mean (SD)) | 41.97 (28.01) | 33.51 (26.65) | 0.129 | 0.309 |
| QOL (mean (SD)) | 39.16 (21.78) | 33.61 (18.51) | 0.174 | 0.275 |
| Lysholm Score (mean (SD)) | 64.79 (18.36) | 58.54 (19.27) | 0.121 | 0.332 |

SMD, Standardized Mean Difference; SD, Standard Deviation; CCI, Charlson’s Comorbidity Index; AKO, Around-Knee Osteotomy; MOWHTO, Medial Opening Wedge High-Tibial Osteotomy; MOWDTO, Medial Opening Wedge Distal Tuberosity Osteotomy; DLO, Double-Level Osteotomy; LCWDFO, Lateral Closing Wedge Distal Femoral Osteotomy; ICRS, International Cartilage Repair Society; MFC, Medial Femoral Condyle; MTP, Medial Tibial Plateau; LFC, Lateral Femoral Condyle; LTP, Lateral Tibial Plateau; PROMs, Patient-Reported Outcome Measures; KOOS, Knee Injury and Osteoarthritis Outcome Score; ADL, Activities of Daily Living; QOL, Quality of Life

**Online Resource 4**

Supplementary Table 3. Adjusted baseline characteristics after overlap weighting: older patients (>65 years).

|  | Nearly Normal Group (N = 33.39) | Abnormal Group (N = 33.39) | P-value | SMD |
| --- | --- | --- | --- | --- |
| Age (years, mean (SD)) | 72.68 (4.31) | 72.96 (4.03) | 0.691 | 0.067 |
| Sex (male) (n (%)) | 9.6 (28.8) | 8.4 (25.3) | 0.654 | 0.08 |
| Body Mass Index (mean (SD)) | 24.79 (2.80) | 25.34 (2.68) | 0.229 | 0.204 |
| Height (mean (SD)) | 1.56 (0.07) | 1.57 (0.09) | 0.359 | 0.164 |
| Weight (mean (SD)) | 60.43 (8.74) | 62.99 (10.52) | 0.145 | 0.265 |
| CCI (mean (SD)) | 0.21 (0.52) | 0.28 (0.48) | 0.397 | 0.14 |
| AKO Type |  |  | 0.682 | 0.207 |
| MOWHTO (n (%)) | 23.5 (70.4) | 21.2 (63.4) |  |  |
| MOWDTO (n (%)) | 4.0 (12.0) | 3.6 (10.9) |  |  |
| DLO (LCWDFO-MOWHTO) (n (%)) | 3.4 (10.1) | 3.1 (9.4) |  |  |
| DLO (LCWDFO-MOWDTO) (n (%)) | 2.5 (7.5) | 5.4 (16.3) |  |  |
| Concomitant Surgical Procedures |  |  |  |  |
| Lateral Retinacular Release (n (%)) | 24.8 (74.2) | 24.8 (74.2) | 1 | <0.001 |
| Cruciate Ligament Reconstruction (n (%)) | 0 (0%) | 0 (0%) | N/A | <0.001 |
| Meniscectomy (n (%)) | 22.4 (67.1) | 21.9 (65.5) | 0.851 | 0.033 |
| Meniscal Repair (n (%)) | 0.6 (1.7) | 0.6 (1.8) | 0.969 | 0.007 |
| Cartilage Repair Methods |  |  |  |  |
| Bone Marrow Stimulation (n (%)) | 24.2 (72.5) | 28.8 (86.4) | 0.06 | 0.349 |
| Osteochondral Autograft Transfer System (n (%)) | 32.4 (97.0) | 30.6 (91.7) | 0.139 | 0.229 |
| Autologous Chondrocyte Implantation (n (%)) | 0.0 (0.0) | 0.0 (0.0) | 0.327 | <0.001 |
| Period from Initial Surgery to Second-Look Arthroscopy (days) (mean (SD)) | 430.57 (55.18) | 451.89 (80.36) | 0.08 | 0.309 |
| Chondral Defect Location at First-look Arthroscopy (ICRS Grade ≥ 3) |  |  |  |  |
| MFC (n (%)) | 32.0 (96.0) | 33.4 (100.0) | 0.089 | 0.29 |
| MTP (n (%)) | 26.4 (79.0) | 29.5 (88.5) | 0.166 | 0.259 |
| LFC (n (%)) | 1.1 (3.1) | 7.0 (20.9) | <0.001 | 0.568 |
| LTP (n (%)) | 0.1 (0.3) | 3.3 (9.9) | <0.001 | 0.449 |
| Patella (n (%)) | 7.0 (21.1) | 17.3 (51.9) | <0.001 | 0.676 |
| Trochlea (n (%)) | 14.2 (42.4) | 24.5 (73.4) | <0.001 | 0.66 |
| Chondral Defect Location at Second-look Arthroscopy (ICRS Grade ≥ 3) |  |  |  |  |
| MFC (n (%)) | 12.8 (38.3) | 18.0 (54.0) | 0.068 | 0.32 |
| MTP (n (%)) | 20.4 (61.1) | 22.1 (66.3) | 0.544 | 0.108 |
| LFC (n (%)) | 0.1 (0.2) | 3.5 (10.5) | <0.001 | 0.472 |
| LTP (n (%)) | 0.1 (0.3) | 4.1 (12.2) | <0.001 | 0.505 |
| Patella (n (%)) | 2.7 (8.0) | 19.5 (58.5) | <0.001 | 1.268 |
| Trochlea (n (%)) | 0.0 (0.0) | 33.4 (100.0) | <0.001 | NaN |
| Pre-Operative PROMs |  |  |  |  |
| KOOS subscales |  |  |  |  |
| Pain (mean (SD)) | 59.65 (17.57) | 58.26 (18.94) | 0.662 | 0.077 |
| Symptoms (mean (SD)) | 66.31 (19.16) | 59.65 (18.37) | 0.04 | 0.355 |
| ADL (mean (SD)) | 73.29 (15.18) | 72.59 (14.06) | 0.778 | 0.048 |
| Sports (mean (SD)) | 34.54 (23.20) | 34.54 (25.69) | 1 | <0.001 |
| QOL (mean (SD)) | 39.55 (22.79) | 40.71 (19.02) | 0.742 | 0.055 |
| Lysholm Score (mean (SD)) | 60.97 (20.44) | 58.85 (17.35) | 0.506 | 0.112 |

SMD, Standardized Mean Difference; SD, Standard Deviation; CCI, Charlson’s Comorbidity Index; AKO, Around-Knee Osteotomy; MOWHTO, Medial Opening Wedge High-Tibial Osteotomy; MOWDTO, Medial Opening Wedge Distal Tuberosity Osteotomy; DLO, Double-Level Osteotomy; LCWDFO, Lateral Closing Wedge Distal Femoral Osteotomy; ICRS, International Cartilage Repair Society; MFC, Medial Femoral Condyle; MTP, Medial Tibial Plateau; LFC, Lateral Femoral Condyle; LTP, Lateral Tibial Plateau; PROMs, Patient-Reported Outcome Measures; KOOS, Knee Injury and Osteoarthritis Outcome Score; ADL, Activities of Daily Living; QOL, Quality of Life
